# Sex differences of anthropometric indices of obesity by age among Iranian adults in northern Iran: A predictive regression model

**Published:** 2015

**Authors:** Karimollah Hajian-Tilaki, Behzad Heidari

**Affiliations:** 1 Department of Biostatistics and Epidemiology, Babol University of Medical Sciences, Babol, Iran.; 2 Department of Internal Medicine, Ayatollah Rouhani Hospital, Babol University of Medical Sciences, Babol, Iran.

**Keywords:** Body mass index, Waist circumference, Waist to hip ratio, Quadratic form, Adults

## Abstract

**Background:**

Background and Objectives: The biological variation of body mass index (BMI) and waist circumference (WC) with age may vary by gender. The objective of this study was to investigate the functional relationship of anthropometric measures with age and sex.

**Methods:**

The data were collected from a population-based cross-sectional study of 1800 men and 1800 women aged 20-70 years in northern Iran. The linear and quadratic pattern of age on weight, height, BMI and WC and WHR were tested statistically and the interaction effect of age and gender was also formally tested.

**Results:**

The quadratic model (age^2^) provided a significantly better fit than simple linear model for weight, BMI and WC. BMI, WC and weight explained a greater variance using quadratic form for women compared with men (for BMI, R^2^=0.18, p<0.001 vs R^2^=0.059, p<0.001 and for WC, R^2^=0.17, p<0.001 vs R^2^=0.047, p<0.001). For height, there is an inverse linear relationship while for WHR, a positive linear association was apparent by aging, the quadratic form did not add to better fit.

**Conclusion:**

These findings indicate the different patterns of weight gain, fat accumulation for visceral adiposity and loss of muscle mass between men and women in the early and middle adulthood.

General obesity defined by high body mass index (BMI) and abdominal obesity defined as high waist circumference (WC) or high waist to hip ratio to be major public health problem in both developed and developing countries (1-4). A significant increasing of these indexes was essentially occurred with high caloric dietary habits and low physical activity level. It is well-established that BMI and WC are independent predictors of several cardiovascular risk factors (5) and morbidity (6, 7). In particular, higher WC associated with greater level of visceral adiposity and those with a high WC, has a greater chance of mortality of chronic diseases such as type 2 diabetes and cardiovascular diseases (8-10). In recent decades, the worldwide epidemic of obesity and central obesity has continued to be extended in developing countries (11-16). In particular, Iranian population experienced an increased demographic changes and epidemiologic transition over the past three decades (3, 4). Improving the economic status and modernization of society changed the lifestyles leading to an acceleration in the consumption of high caloric foods and the sedentary behavior with subsequent increase in body mass index (BMI) and waist circumference (WC) in adults population (2, 3, 4, 16) and thus, high prevalence of obesity and central obesity became the major public health problems (2-4, 16).

In this context, the causes of death have been shifted from infectious diseases to non-communicable chronic diseases such as cardiovascular diseases and cancers in Iran. By aging, the fat accumulation in adulthood leads to an accelerated BMI and WC in the elderly with a maximum age of almost 60 years and the loss of muscle mass starts in middle adulthood and continues to old age (17).

Overall, BMI and WC have been influenced by several socio-demographic characteristics in different societies. Their pattern differs with age, gender and other socio-economic factors. The biological variation and association of BMI and WC with age may have different patterns of effect and even it may vary by gender. In this regard, there are few studies in large and representative samples of adult population of Iran. 

With a representative sample of adult population living in four large cities in northern Iran, we addressed the following three questions: 1) what proportion of weight, BMI, WC and WHR variances can be explained by age? 2) What are the shapes of these functional relationships in terms of linear and quadratic forms? 3) Does the functional relationship with age vary by gender status?

## Methods

**Study subjects and population: **Study subjects were recruited from our provincial household survey of obesity assessment in a representative sample of population-based cross-sectional study in the North of Iran in 2004. The study design, population and sampling procedure were described previously in details (4). Data were collected from 1800 men and 1800 women, aged 20-70 years, apparently the healthy subjects with no known debilitating or systematic diseases were residence of four large cities in south of Caspian sea. The demographic data such as age and sex were extracted. The protocol was approved by Research and Ethics Committee of Babol University of Medical Sciences. The written informed consent was obtained from all subjects prior to their participation in the study.

**Anthropometric Measurements: **Height was measured with subject standing barefoot with heels together, arms legs straight and shoulders relaxed and scale to the nearest 0.1 cm and weight was taken to nearest 0.1 kg with a SECA platform scale graduated to the nearest 0.1 kg with subjects standing on platform barefoot. From these two measures, body mass index (BMI) was calculated by weight in kilogram divided by square of height in meter (kg/m^2^). WC was measured on waist diameter at the level of midpoint between iliac crest and lower border of tenth rib. Hip was defined as the widest circumference around the buttocks below the iliac crest Waist to hip ratio (WHR) was calculated as the waist circumference divided by the hip circumference (both in centimeters). SPSS software was used for statistical analysis. 

First, the age-sex descriptive statistics were summarized by means (±SD) with 95% confidence intervals (CI) for different measures. Then, the patterns of weight, height, BMI, WC and WHR with age were assessed by linear regression models and the linear and quadratic effect of age on weight, height, BMI, WC and WHR were tested statistically and the interaction effect of age and gender was formally tested in regression model. The linear model was built with respect to gender for predicting anthropometric measures with age and a p-value less than 5% was considered as significant level.

## Results

The mean (±SD) ages were 38.5±14.3 and 37.5±13.0 years for men and women, respectively. Table 1 presented the mean ± SD of anthropometric measurements specific for sex and age groups. Men were heavier and taller than women in all age groups (p<0.001). 

Weight showed a significant increase with age up to 50 years in both sexes and then tends to decrease. An apparent decline of the mean of height with aging was observed in both sexes. The average of BMI values were higher in women than men in all age groups significantly (p<0.001) with apparent decline of BMI at age of 50 y for men and 60 y for women. 

The slopes of BMI for increasing with age at 20 to 50 years was greater of magnitude in women than and men; the slope of decline trend at age 60 and later was also accelerated in women. Waist circumference also increased significantly with age up to 60 years in both sexes (p<0.001). A rapid increase of slope in at the side WC was observed in early adulthood for women compared with men. Overall, the mean of WC was significantly higher in men than women but the differences were not significant in age group of 40-49 years and 60-70 years. 

**Table 1:** The frequency and age-sex specific of mean ± SD of anthropometric measures and p-value

**Anthropometric measures**	**Age groups (year)**	**Men (n=1800)**		**Women (n=1800)**		**P-value**
		**n**	**Mean± SD**	**N**	**Mean ±SD**	
Weight (kg)	20-29 30-39 40-49 50-59 60-70 Total	620 392 347 254 196 1800	72.5±12.0 76.5±11.7 75.5±11.4 74.8±11.8 70.1±12.4 74.0±12.1	608 447 373 256 118 1800	62.7±12.9 69.6±11.8 72.7±12.3 72.3±12.8 67.8±12.2 68.2±13.1	<0.001 <0.001 <0.001 0.02 0.12 <0.001
Height (cm)	20-29 30-39 40-49 50-59 60-70 Total	619 391 374 245 196 1798	174.5±7.4 173.1±7.2 170.9±7.4 170.4±7.7 167.5±8.2 172.2±7.8	608 446 372 256 118 1798	161.1±6.2 159.9±6.4 158.3±6.5 157.8±6.8 154.5±7.6 159.3±6.7	<0.001 <0.001 <0.001 <0.001 <0.001 <0.001
BMI(kg/m^2^)	20-29 30-39 40-49 50-59 60-70 Total	619 391 474 245 196 1798	23.8±3.8 25.6±3.8 25.9±3.8 25.8±3.7 24.9±4.0 25.0±3.9	608 446 372 256 118 1798	24.2±4.7 27.3±4.8 29.1±4.9 29.1±5.2 28.5±5.2 26.9±5.4	0.14 <0.001 <0.001 <0.001 <0.001 <0.001
WC (cm)	20-29 30-39 40-49 50-59 60-70 Total	620 392 346 245 196 1799	86.1±10.6 89.8±11.8 91.5±10.6 91.9±11.9 91.0±12.4 89.3±11.5	607 447 373 254 118 1799	81.0±11.8 87.9±12.2 92.1±12.4 94.9±13.2 92.8±13.2 87.8±13.5	<0.001 0.02 0.46 0.009 0.23 <0.001
WHR	20-29 30-39 40-49 50-59 60-70 Total	620 392 346 245 196 1799	0.87±0.09 0.88±0.12 0.89±0.09 0.91±0.12 0.92±0.09 0.89±0.11	608 446 373 253 118 1798	0.81±0.11 0.82±0.08 0.84±0.09 0.86±0.09 0.87±0.09 0.83±0.09	<0.001 <0.001 <0.001 <0.001 <0.001 <0.00

SD: standard deviation, BMI: body mass index, WC: waist circumference, WHR: waist to hip ratio

**Figure 1:**
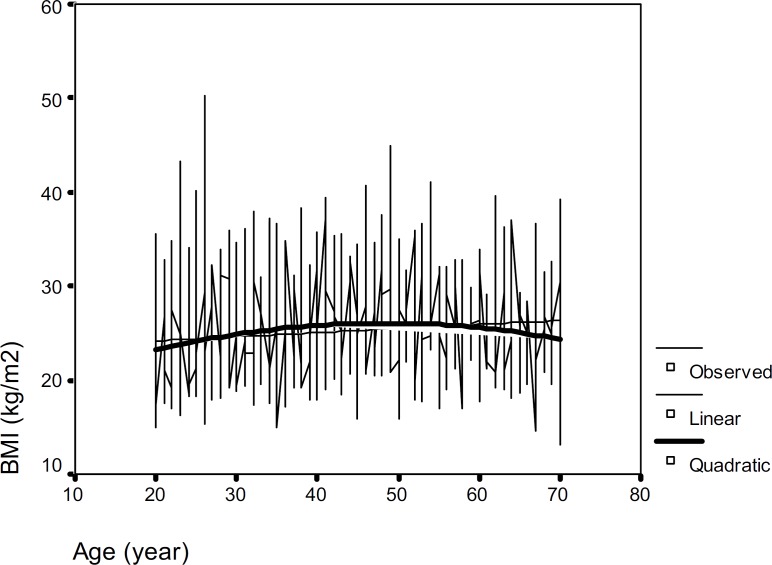
The BMI pattern with age in men

**Figure 2:**
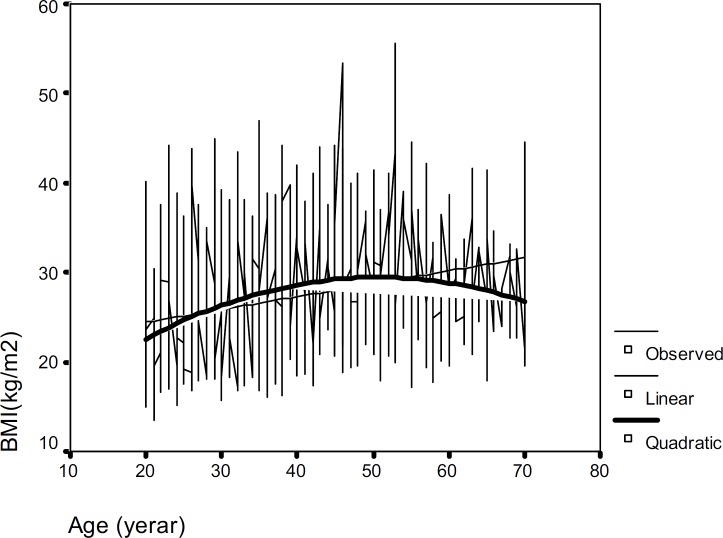
The BMI pattern with age in women

The mean of WHR was significantly higher in men compared with women and also an apparent increase of WHR was observed with age in both sexes. Figures 1 and 2 show the observed plot and the linear and quadratic patterns of BMI versus age for men and women, respectively. Table 2 shows sex-specific intercepts and slopes for the changes of weight, height, BMI, WC and WHR with aging. In both sexes, the quadratic model, including age^2^ provided a significantly better fit than simple linear model for BMI and waist circumference. BMI and WC explained greater variance in quadratic model for women compared with men (for BMI, R^2^=0.18, p<0.001 vs R^2^=0.06, p <0.001 and for WC, R^2^=0.17, p<0.001 vs R^2^=0.05, p<0.001). The slopes of quadratic age-related decline were significant in men and women. 

The slopes with age in average values of both BMI and WC were of greater magnitude in women compared with men. A significant age-sex interaction effect was detected for both WC and BMI i.e. the changes of slopes of BMI and WC with age varies with sex significantly and a greater positive linear slope and negative slope for quadratic terms were observed for women.

Regression models for weight versus age, the quadratic model including age^2^ provided a significant better fit than the simple linear model in both sexes but weight explained a greater variance using the quadratic model for women than men (R^2^=0.13 vs R^2^= 0.03, respectively). While for height, an inverse linear relationship (negative slope) was apparent by aging and the quadratic form did not add an additional fit on the magnitude of R^2^ in both sexes. 

In addition, regression model was built for WHR, a positive linear association was revealed by aging and quadratic term did not prompt an additional gain for better fit in terms of R^2^.

**Table 2:** The regression coefficient of linear and quadratic effect of age on anthropometric measures, R^2^ and P-value with respect to gender

**Sex**	**Dependent variables**	**Model**	**Intercept**	**Slope (age)**	**Slope (age** ^2^ **)**	**R** ^2^	**P-value**
Men	Weight	Linear Quadratic	74.32 57.32	0.009 0.91	- -0.01	0.00 0.03	0.65 <0.001
	Height	Linear Quadratic	178.4 177.87	-0.16 -0.13	- -0.0004	0.09 0.09	<0.001 <0.001
	BMI	Linear Quadratic	23.3 17.6	0.04 0.35	- -0.0036	0.025 0.059	<0.001 <0.001
	WC	Linear Quadratic	83.45 73.88	0.15 0.67	- --0.006	0.035 0.047	<0.001 <0.001
	WHR	Linear Quadratic	0.84 0.89	0.001 -0.002	- -0.0003	0.02 0.03	<0.001 <0.001
Women	Weight	Linear Quadratic	58.99 30.44	0.24 1.81	- -0.019	0.06 0.13	<0.001 <0.001
	Height	Linear Quadratic	164.4 163.5	-0.14 -0.08	- -0.0007	0.07 0.07	<0.001 <0.001
	BMI	Linear Quadratic	21.57 10.68	0.14 0.74	- -0.007	0.12 0.18	<0.001 <0.001
	WC	Linear Quadratic	73.49 53.3	0.38 1.49	- -0.013	0.13 0.17	<0.001 <0.007
WHR	Linear Quadratic	0.77 0.77	0.001 0.001	- -	0.04 0.04	<0.001 <0.007

## Discussion

The present study provided statistical model for obesity related anthropometric indexes in a relative large representative sample in Iranian adults in northern Iran. Our findings show that the quadratic model, including age^2^ provided a significantly better fit than simple linear model for weight, BMI and waist circumference. A greater magnitude of variance of weight, BMI and WC was explained by quadratic model for women compared with men. A significant age-sex interaction effect was detected for weight, BMI and WC i.e. the changes of slopes of age varies with sex significantly and a greater positive linear slope and negative slope for quadratic terms were observed for women.

Quadratic terms including age^2 ^indicated a possible greater acceleration in changing of slopes of obesity-related indexes such as weight, BMI and WC with aging for women compared with men. In women, the slope of BMI was sharper at age of 30-39 years and it tends to decline at age 50-59 years. Then, it switches to be negative slope at age near to 60 years. A similar pattern of slopes for weight and WC as apparent and the switching points were again at age almost 60 years and also the change in declining slope was greater for women than men. In men, in early adulthood, the slope is not as sharp as of women and the changes of switching occurred at age near 50 years. The sharper pattern of slopes of obesity-related indices in women at early and middle adulthood, can be explained by parity, low physical activity and low education level (4). 

The positive association of age with total adipose tissue was also found in both genders aged 38-60 years by Oka et al. (18). In another study among Chilean adults, aged 60-99 years, the decline of mean of BMI, weight and WC was observed with age and the degree of decline in slope was also greater of magnitude for women than men (17). However, they reported that the quadratic terms related to decline with age were not statistically significant in men for any anthropometric indices studied. This dissimilarity is related to the range of age of subjects in a Chilean study that was quite different from ours. The rapid changes in slopes of obesity-related indexes in elderly with age in women can be explained by the changes of dietary pattern toward a deficient consumption of most relevant nutrients with significant lower intake of energy, protein, calcium, iron and folic acid in elderly. In a study by Woo et al. in Chinese adults aged 20-88 years, in both sexes, height and lean mass decreased in a linear fashion with age and changes in weight, BMI and fat mass and percentage of fat with aging were also present in women and followed a quadratic trend (19) that is similar to our results. In contrast, in another study by Chen et al, in Beijing, BMI increased gradually from 21.1 kg/m2 in ages 20-29 years to 26.1 kg/m2 in ages 70-74 years in women (20). While in our findings, the baseline BMI (24.2Kg/m2) of women in ages 20-29 years was higher and the trend with age differs than those reported in Chinese women (20). Based on our findings, the quadratic model by age alone explained 18%, 17% and 13.0% of variances of BMI, WC and weight among women, respectively. In contrast, Welch and Sowers reported that the quadratic model of age alone accounted for 8% of observed variance in fatness in women aged 18-94 years (21). 

In our study, an inverse linear relationship (negative slope) and a linear positive association with age and height and WHR was respectively apparent for both genders and the quadratic form did not add a better fit as determined by R^2^ in estimating height and WHR in both sexes. Among the Chinese adults, aged 20-88 years, in both sexes, height and lean mass decreased in a linear fashion with age. The reduction of average of height with age estimated in our study was also consistent with that reported by Santos et al in Chilean study in elderly (17). 

This reduction can be explained by cohort effect due to secular trend for differences in stature because of changing environmental conditions toward improving nutritional status of younger age group in epidemiologic transition. While cross-sectional nature of design of this study restricts to establish clearly this secular trend, similar height changes were also present in other settings studied (17, 22).

 A limitation of this study is the design of the study which is cross-sectional, restricting the inferences with regard to body composition changes with aging and it is not adequate for examining the temporal sequence of aging and changing in anthropometric measures and also the secular trend can not be established clearly. 

In addition, due to cross- sectional nature of this study, an interpretation of estimated slopes in terms of decline/increase must be performed with caution. Thus, we can only say the apparent changes of slope for anthropometric indexes by age.

In conclusion, our data based on a large representative sample of subjects, suggest that the changes of BMI and WC associated with age are of greater magnitude in women than men indicating the different patterns of weight gain, fat accumulation for visceral adiposity and loss of muscle mass between men and women in the early and middle adulthood.
